# Reappraising the psychosomatic approach in the study of “chronic orofacial pain”: looking for the essential nature of these intractable conditions

**DOI:** 10.3389/fpain.2024.1349847

**Published:** 2024-05-10

**Authors:** Akira Toyofuku, Hirofumi Matsuoka, Yoshihiro Abiko

**Affiliations:** ^1^ Department of Psychosomatic Dentistry, Graduate School of Medical and Dental Sciences, Tokyo Medical and Dental University (TMDU), Tokyo, Japan; ^2^ Division of Disease Control and Molecular Epidemiology, Department of Oral Growth and Development, School of Dentistry, Health Sciences University of Hokkaido, Ishikari-Tobetsu, Japan; ^3^ Division of Oral Medicine and Pathology, School of Dentistry, Health Sciences University of Hokkaido, Ishikari-Tobetsu, Japan

**Keywords:** burning mouth syndrome, atypical odontalgia, psychosomatic oral pain, chronic orofacial pain, treatment strategy

## Abstract

As burning mouth syndrome (BMS) and atypical odontalgia (AO) continue to remain complex in terms of pathophysiology and lack explicit treatment protocol, clinicians are left searching for appropriate solutions. Oversimplification solves nothing about what bothers us in clinical situations with BMS or AO. It is important to treat a complicated phenomenon as complex. We should keep careful observations and fact-finding based on a pragmatic approach toward drug selection and prescription with regular follow-up. We also need to assess the long-term prognosis of treatment with a meticulous selection of sample size and characteristics. Further investigation of BMS and AO from a psychosomatic perspective has the potential to provide new insight into the interface between brain function and “chronic orofacial pain.”

## 1 Introduction

“Chronic orofacial pain (COFP)” is an umbrella term used to describe painful regional syndromes with a chronic, unremitting pattern (1). This term is very convenient; however, the author does not prefer the term “chronic orofacial pain” due to its lack of therapeutic indications and potential for confusion (2). In fact, the study of COFP now seems to be losing focus because of its ambiguity.

For example, studies on burning mouth syndrome (BMS) have seen remarkable growth in the last two decades. These study data have many limitations and do not apply to many clinical cases. Management of BMS has been seen as a “jumble of wheat and tares,” with little evidence to support or refute interventions. The existence of “too many reviews and too few trials” leads to difficulty in choosing an appropriate therapy for each patient with BMS (3).

Consequently, BMS often persists for many years, and patients may undergo several unproductive tests without any improvement in oral symptoms despite many treatment attempts (4). Dentists obviously feel the urgent need to offer some treatments for these BMS patients, developing a feeling of helplessness and frustration.

Atypical odontalgia (AO) is included as another COFP condition that presents challenges for many dentists (5). This pain condition has been given more attention by many dentists because conventional dental procedures seldom provide relief for these patients; on the contrary, there is a risk of legal troubles. AO “pain” differs significantly from ordinary dental conditions like caries or pulpitis; however, patients’ complaints are sometimes very confusing to distinguish from such ordinary dental pain that can be treated successfully. Dentists have become more cautious and nervous in diagnosing “toothache” and more careful when performing invasive dental procedures these days.

Moreover, confusing terminology is impeding progress in the research for the treatment and pathophysiology of both conditions. It might be accurate to say that there is no perfect treatment that can be effective for all BMS or AO patients with various underlying backgrounds. In my opinion, the lack of “psychosocial interventions” is probably the most critical factor contributing to this confusing situation.

In this brief opinion article, BMS and AO are mainly argued as “psychosomatic oral pain”; on the other hand, temporo-mandibular disorders (TMDs) and trigeminal neuralgia (TN) are distinguished from them.

## 2 What type of “pain” are patients complaining about in cases of BMS or AO?

Many studies have indicated the important role of psychological factors such as depression and anxiety in BMS and AO. Nonetheless, most of them have remained superficial, failing to suggest any hopeful solutions for these chronic oral pain conditions. It seems nonsensical to argue the efficacies of antidepressants or other neuromodulations for BMS or AO without accompanying “psychosocial interventions.” Like other chronic pain, treatment outcomes of BMS were affected easily by placebo and nocebo effects (6). Therefore, every treatment outcome of BMS and AO is probably affected by the patient–physician relationship. Moreover, the patient–physician relationship is crucial for patient’s adherence to any pain medications.

Mere administration without a convincing reason and a full understanding of patients would easily result in their non-adherence. The patient–physician relationship is one of the biggest watersheds between adherence and non-adherence. It should be prioritized to be aware of this psychological background underlying every prescription. Pain medication for BMS and AO requires this psychosomatic perspective. “Psychoeducational treatment” (Table 1) would be necessary for successful pain medication. This is one of the very basic cognitive behavioral therapies (7).

**Table 1:** Psychoeducation before pain medication.

1. Careful explanations of the pathophysiological model of pain Relationship between central sensitivity and chronic oral pain with unknown origin Not merely “psychogenic” but hypersensitivity of the brain 2. Getting to understand and agree on the treatment goal Confirm target of medication, Data on the efficacy of antidepressants, Possible side effects, Needs for continuation of at least 6 months 3. Behavioral activation Regularly rhythmical daily life; Enough sleep, a healthy diet, and light exercise Balance between rest and action, Monitoring (e.g., pain dialy), Pacing (time-contingent approach)

BMS and AO share common trigeminal nerve input, yet they are highly distinct disorders (8). Somatotopic segregation may occur at the level of the trigeminal nucleus, thalamus, and somatosensory cortex, and distinct ionic or neurochemical signaling pathways may be involved (9). This structural basis probably has a strong connection with instinctual emotional function, easily affected by various psychosocial factors.

BMS and AO might be seen as models of a psychosomatic disorder, in which the biological environment interacts with psychosocial factors. This approach does not mean that the mechanisms underlying BMS and AO are purely psychological, but that the role of psychological (or psychopathological) factors is more substantial than in most diseases (4).

In Japanese dentistry, BMS and AO have been regarded as oral psychosomatic disorders for more than half a century, requiring a multidisciplinary (medical and psychosocial) approach. Amitriptyline, a classic tricyclic antidepressant (TCA), has been used for both BMS and AO, with the need for accompanying psychotherapies since then. Nevertheless, difficulties in time-consuming psychosomatic treatments and poor reimbursement (healthcare fee) have prevented many dentists from diligent practice for such patients. However, we have kept searching for BMS and AO as “psychosomatic oral pain” in the hope of finding treatments for them.

## 3 Problems pile up in researching BMS and AO

### 3.1 Heterogeneity

The heterogeneity of BMS or AO is the biggest barrier preventing us from reaching the best treatment (3). Moreover, BMS symptoms may change fluidly over time. Sometimes, burning pain goes successfully; however, relapse of oral discomforts such as xerostomia or taste disturbances might quickly become a new problem instead of pain.

The nature of BMS is precisely that of a syndrome, which has several causative factors, including the psychosomatic nature of chronic pain. Hence, treatment response might differ depending on the predominance of individual confounding pathological factors such as neuropathic component, central sensitization, or psychiatric comorbidities. The problems are intertwined in so complex a way that they cannot be solved completely by a single therapy (3).

In particular, psychiatric comorbidities might be significant for any treatments of both BMS and AO. Specifically, when planning pharmacotherapy, one should always consider the psychiatric condition and involve a complete psychologic/psychiatric assessment (10).

Recently, we have had to pay more attention to neurodevelopmental disorders hidden behind intractable AO or BMS (11, 12). Their hypersensitivity might make the pain treatments more difficult; however, treatment response for a dopaminergic medication suggests some common pathophysiology underlying both conditions (13). Regarding these clinical phenomena, confirming pharmacological response (e.g., TCA-responsive BMS/AO vs. non-responders) is one of the challenging issues in understanding the pathophysiology of this pain (14).

On the other hand, neurovascular compression of the trigeminal nerve might also be valuable to distinguish possible peripheral pathophysiology of AO (15).

### 3.2 Oral cenesthopathy superimposed on BMS or AO

The complaint of “burning” is often regarded as neuropathic pain; however, it also has a very similar nature to oral cenesthopathy (16). Oral cenesthopathy is characterized by bizarre and abnormal oral sensations without medical and dental evidence. In fact, oral cenesthopathy is sometimes comorbid with BMS (26.24%) or AO (5.78%) (17).

The diagnosis of oral cenesthopathy is still controversial, and contemporary psychiatry does not provide independently defined diagnostic criteria (18). Oral sensory disturbances fall within a continuum in patients with or without diagnosed somatoform disorders. Careful consideration of the patient's dopaminergic state and the possible contribution of psychiatric comorbidities can help guide therapeutic choices, but the management may still involve some trial and error since symptoms evolve and overlap (19).

### 3.3 Assessment of improvement

In chronic pain research like BMS or AO, the biggest problem remains in how to assess subjective oral symptoms that cannot be quantitated. Next, what should be set as the treatment goal or target? How can we say a patient with BMS or AO has been saved?

A satisfactory assessment tool for BMS remission is not yet available. The suffering of BMS or AO could hardly be assessed in visual analog scale (VAS) scores only. BMS involves not only a burning sensation but also discomfort such as dryness or dysgeusia (20), as mentioned above. Therefore, the clinicians should reconsider what a patient claims as “pain.” We need more effective qualitative assessment tools for insight into the patient’s experience of “pain” instead of using VAS only.

A standardized symptom assessment tool is necessary to facilitate scientific discussion among researchers for improving diagnosis and treatment modalities. We developed the Oral Dysesthesia Rating Scale (Oral DRS) and evaluated its validity as an assessment tool (18). Since patients often develop impairments in oral functions such as eating and speaking and in the performance of daily activities, this new tool is designed to also assess these dysfunctions.

We believe that the treatment goal or target for BMS or AO should not be set in “complete remission” nor “symptom-free” but good enough satisfaction for both patients and physicians. It must be hastened to develop better “clinically meaningful outcomes.”

### 3.4 Safety of pharmacotherapy

Despite no strong evidence of the efficacy of specific medications or agreement between the authors, it is worth noting that the absence of evidence is not evidence of absence. Neuromodulators such as benzodiazepines (e.g., clonazepam) or antidepressants (e.g., amitriptyline) have been used for the treatment of BMS (21) or AO (14). We have anecdotal evidence in many patients that these drugs work well.

These medication therapies can be continued as long as the patient's benefits outweigh the harm. Tricyclic antidepressants are not always safe (22), and there is the risk of abuse with benzodiazepines (23). However, in Japan, we seldom experience big problems such as dependence or misuse in prescribing benzodiazepines for BMS patients (24). It might be due to the different prescription “refill” service systems in each country. However, benzodiazepine therapy should only ever be initiated when the patient is aware of the risks and benefits of these drugs, understands what physiologic dependence is, and has a clear understanding that the drug will be discontinued after a short time (25). Physicians should weigh the risks versus benefits when prescribing benzodiazepines to patients with BMS. A low-dose strategy in these medications is probably appropriate in most cases.

### 3.5 Lack of long-term prognosis

Then, another important problem arises in the assessment of duration and follow-up of medications. BMS and AO have continuous, long-lasting symptoms, often with fluctuations. Despite the importance of studies evaluating the long-term prognosis, there is little data on longitudinal outcomes or recurrence in treating BMS or AO.

We cannot ignore the systemic problem in university hospitals, where many staff members transfer their positions frequently. It becomes challenging for a patient to be followed up by one physician. This unstable treatment situation must be affected by the treatment effect and the dropout ratio.

Patients with BMS or AO tend to easily drop out from any treatment. We believe evaluating the differences between dropout cases and the cases in good clinical courses would help resolve this (26). We suggest that real-world data may be more essential than short-term RCTs to know the best benefits and limitations of the treatment.

Retrospective long-term treatment outcomes may be a more critical option (27, 28). Complete remission of BMS or AO is not so frequent in these medication therapies; however, it is not always impossible if adequate psychosocial intervention is available.

### 3.6 What should we do next?

It might also be helpful to clarify the factors contributing to patient satisfaction with long-term observations. Goal attainment scaling (GAS) (29), a flexible and responsive technique for assessing outcomes in complex interventions, assimilates the achievement of individual goals into a single standardized “goal attainment scale.” GAS has been proposed as a patient-centered, semi-quantitative measure. Each patient's problems are identified through agreement between the physician and the patient. Treatment goals are set for each problem using the specific, measurable, attainable, realistic, and timed (SMART) methodology. Such an assessment method could shed light on a new treatment strategy that reinforces the previous treatments for BMS and AO.

## 4 Summary

As BMS and AO continue to remain complex in terms of pathophysiology and lack explicit treatment protocols, clinicians are left searching for appropriate solutions.

Oversimplification solves nothing about what bothers us in clinical situations with BMS or AO. It is important to treat a complicated phenomenon as complex. We should keep careful observations and fact-finding based on a pragmatic approach for drug selection and prescription with regular follow-up. We also need to assess long-term prognosis of treatment with a meticulous selection of sample size and characteristics (Table 2). Further investigation of BMS and AO with a psychosomatic perspective can provide new insight into the interface between brain function and “COFP.”

**Table 2:** Future perspectives for the study of chronic orofacial pain.

1. Focus on the hopeful treatment strategy rather than classification or terminology.
2. Screening and management of psychiatric comorbidities (e.g., depressive disorder, anxiety disorder, bipolar disorder, neurodevelopmental disorder)
3. Medication selection and titration of optimal dose (including safety use)
4. Developing basic psychosocial interventions

## Data Availability

The original contributions presented in the study are included in the article/Supplementary Material; further inquiries can be directed to the corresponding author.
